# Decorticate and Decerebrate Response during Pain Stimulation. How Do They Occur?

**DOI:** 10.21315/mjms-12-2024-953

**Published:** 2025-02-28

**Authors:** Che Ku Ashraf Helmi Che Ku Mazuan

**Affiliations:** Postgraduate Neurosurgery Resident Year 1, Department of Neurosciences, School of Medical Sciences, Universiti Sains Malaysia, Health Campus, Kelantan, Malaysia

Dear Editor,

Upon reading with interest the manuscript “Pain as a Guide in Glasgow Coma Scale Status for Neurological Assessment” (1), which thoroughly explains the ascending pain pathway and the sensory-motor cortical interconnections in response to pain stimuli.

I would like to further elaborate on the decorticate and decerebrate responses during the examination of a comatose patient after pain response is initiated.

Generally, limb movement is produced by the contraction and relaxation of the extensor and flexor muscles of the limbs. The relaxation and contraction of these muscles are controlled by separate descending pathways and nuclei. The output from these pathways is antagonistic, one pathway opposes the other to produce movement or maintain posture. The pathways are based on the tracts involved. Four tracts, three of which originate from the brainstem: the corticospinal, vestibulospinal, reticulospinal, and rubrospinal tracts.

Of these, the corticospinal and rubrospinal tracts are responsible for the contraction of the flexor muscles. The corticospinal tracts control both proximal and distal flexor muscle contractions, while the rubrospinal tracts primarily control the contraction of the proximal muscles in the upper extremities. In contrast, the vestibulospinal and reticulospinal tracts are responsible for the contraction of the extensor (antigravity muscles). The vestibulospinal tract controls the proximal extensor muscles, while the reticulospinal tract affects both the proximal and distal extensor muscles. In short, the tracts responsible for the contraction of flexor muscles not only produce excitatory innervation to the flexor muscles but simultaneously inhibiting the extensor muscles.

As outlined in (1), sensory-motor integration occurs at two primary level in the brain: at the cortical level (via direct and indirect tracts, including corticocortical connections that relay sensory signals from the posterior parietal cortex and primary sensory cortex) and at the brainstem level via the Ascending Reticular Activating System (ARAS), particularly the pontine and medullary reticulospinal tracts. Output from the sensory-motor cortex regulates the innervation of the corticospinal, reticulospinal (via corticoreticular fibres), rubrospinal (via corticorubral fibres), and cerebellar pathways, which in turn stimulates the vestibulospinal tracts. Meanwhile, output from the ARAS stimulates the cortical regions responsible for wakefulness and awareness, while the pontine reticulospinal tracts and cerebellum, subsequently stimulates the rubrospinal tracts via cerebellorubral fibres. These physiological systems are illustrated in Figures 1a, 1b and 2.

From this understanding, a lesion or injury can be localised during decerebrate and decorticate posturing. Both conditions involve the loss of cortical input and regulation.

In decorticate posturing, the lesion or injury is located just rostral to the superior colliculus. This lesion interrupts cortical stimulation to all the tracts mentioned earlier. However, due to the ARAS, the rubrospinal tracts can still be activated because the excitatory projection to the red nucleus from the cerebellar nuclei is unaffected. This results in contraction of the proximal limb muscles, limited to the upper extremities (2). Meanwhile, stimulation from the ARAS also activates the pontine reticulospinal tracts and the fastigial nucleus in the cerebellum, which stimulates the vestibulospinal tracts and causes contraction of the lower extremity extensor muscles (2, 3). This explanation is summarised in Figure 3. Location of lesion lies above superior colliculus level, causes decorticate posturing. The remaining intact descending pathway during this condition are mentioned in the diagram.

In decerebrate posturing, the lesion or injury extends further down to the level of the tentorial notch. In this case, all descending cortical systems are interrupted, including the red nucleus, rubrospinal, and vestibulospinal tracts. However, the cells of origin for the excitatory and inhibitory components of the reticular formation lie caudal to the level of the lesion, meaning both sets of reticulospinal tracts (pontine and medullary) remain intact. This results in contraction of the extensor muscles in both the upper and lower extremities (2). The location of lesion lies below midcollicular level and may extend further down to the tentorial notch, causes decerebrate posturing. The remaining intact descending pathway during this condition are mentioned in Figure 4.

Figure 5 showing a schematic diagram which summarises the systems and tracts involved in producing movement of limbs. The green arrows 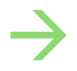 showing the sensory inputs and ascending pathway involved. The red arrows 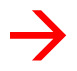 showing the descending tracts and theirs motor outputs. Grey line in A showing example of lesion at superior colliculus level which blocking all the tracts and input above the lesion, resulting in decorticate posturing. Grey line in B showing example of lesion from mid colliculus level and may extend further down to the tentorial notch which blocking all tracts and input including ARAS stimulation to the cerebellum, leaving only reticulospinal tracts remain intact and resulting decerebrate posturing. In conclusion, understanding the mechanisms involved in decorticate and decerebrate posturing during the assessment of an unconscious patient enhances clinical judgement and management, necessitating prompt intervention in acute or emergency settings.

## Figures and Tables

**Figure 1a:**
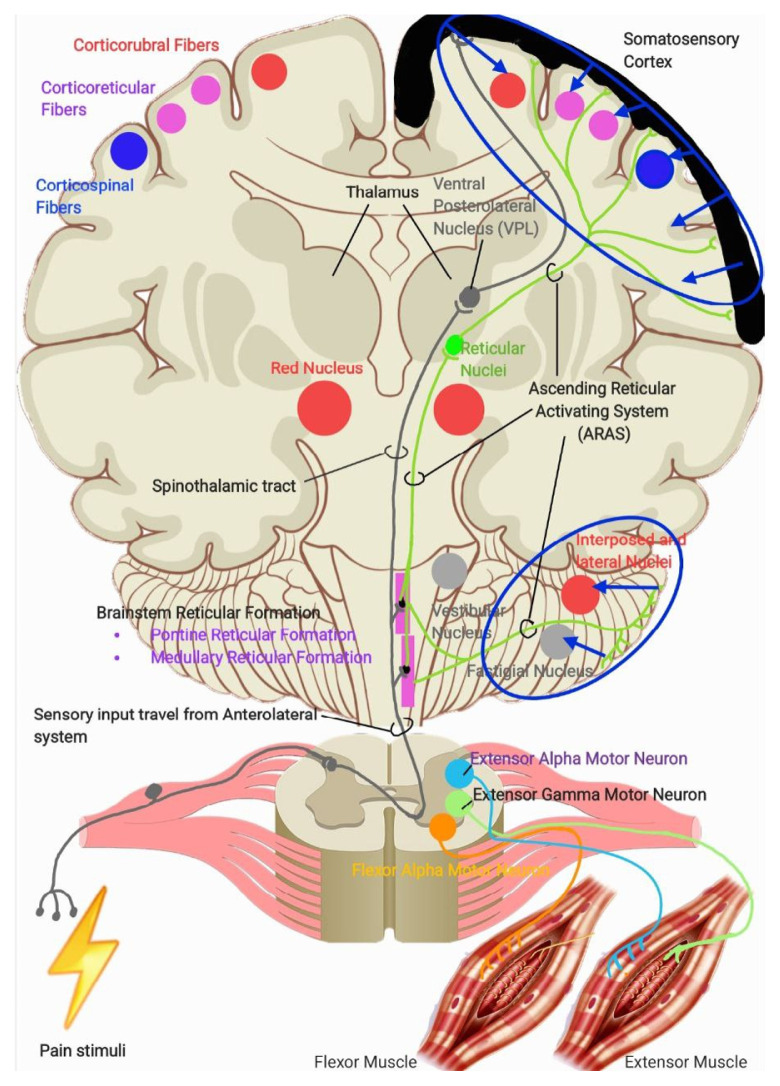
Figure 1a showing the ascending pathway and brief understanding of sensory-motor integration (blue oval with blue arrow)

**Figure 1b:**
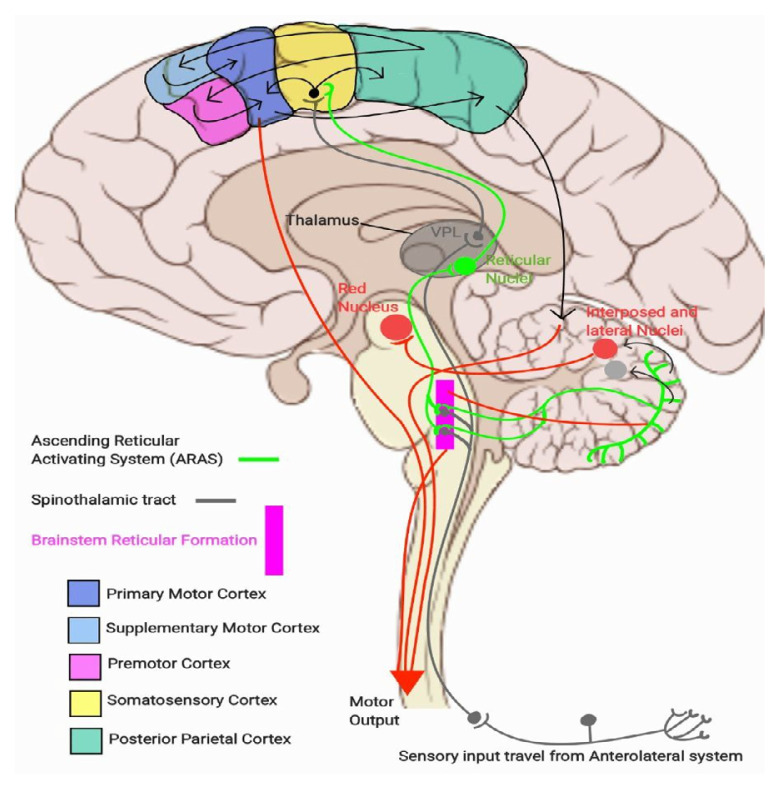
The ascending pathway from saggital cross section and brief understanding of sensory-motor integration (black arrow)

**Figure 2:**
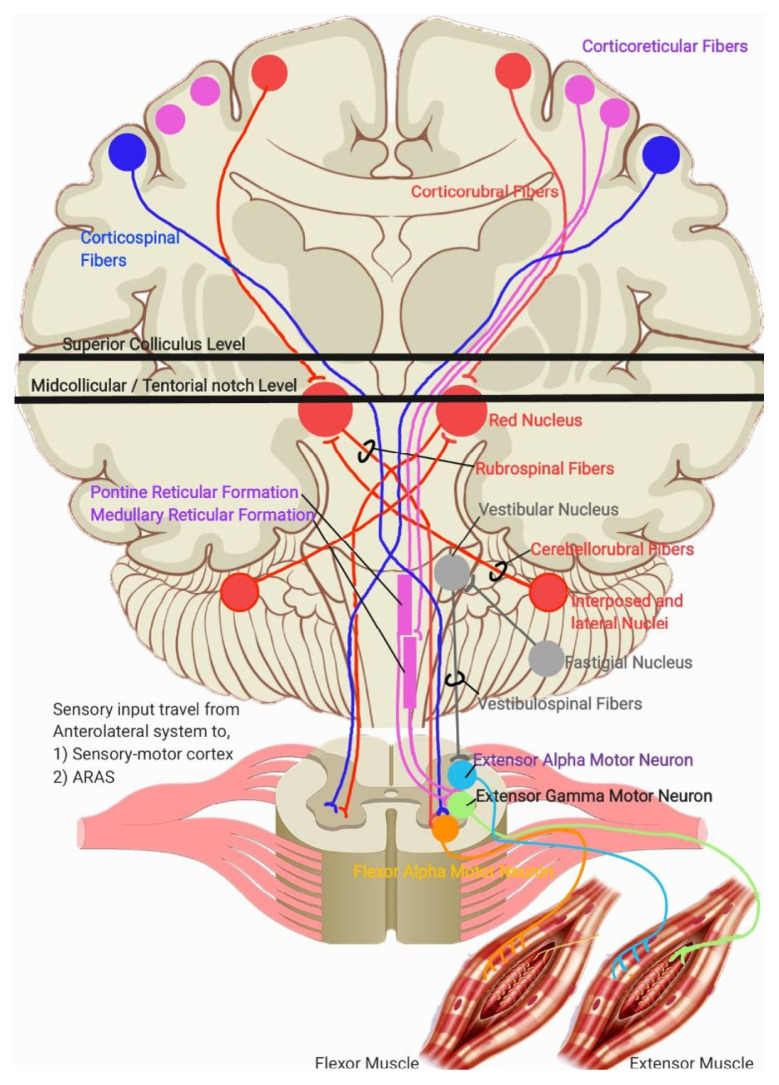
An overview of descending pathways that responsibles to produce movement or maintain posture

**Figure 3:**
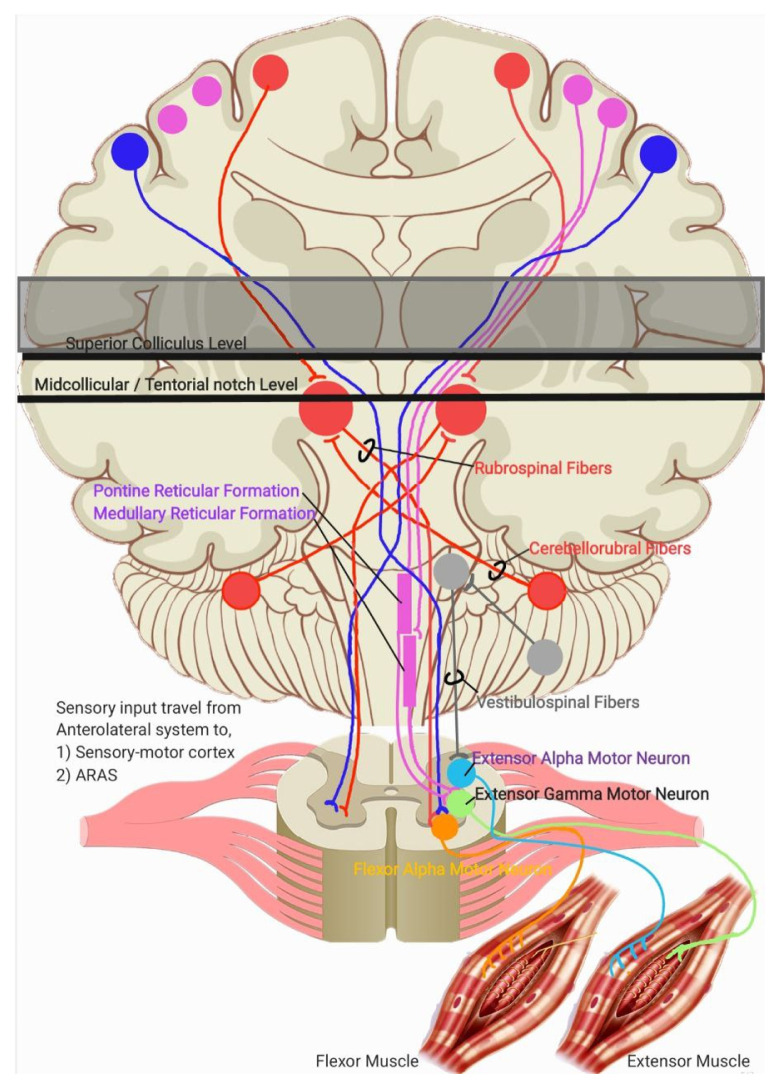
Location of lesion (grey line) that lies above superior colliculus level

**Figure 4:**
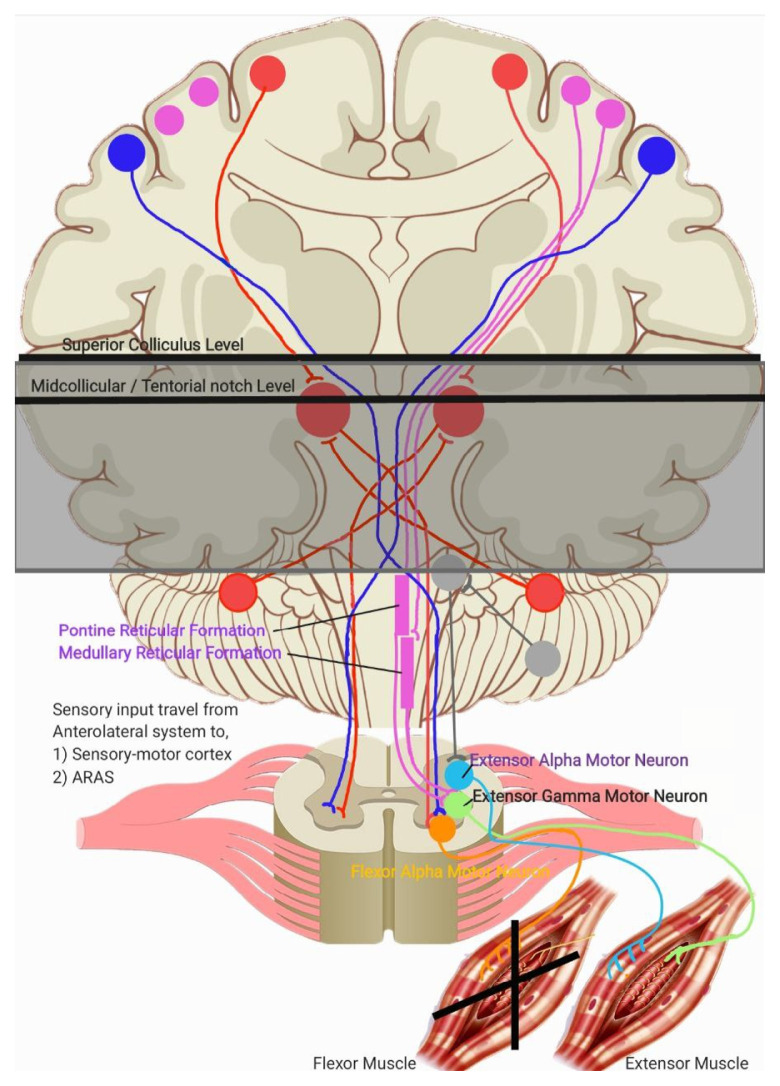
Location of lesion (grey line) that lies below midcollicular level

**Figure 5:**
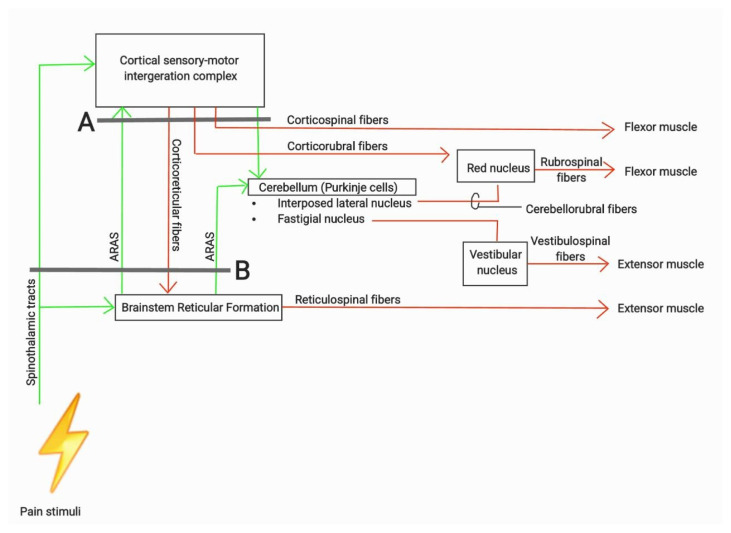
The systems and tracts involved in producing movement of limbs
